# Beyond static risk scores: dynamic world models simulating patient-specific trajectories to inform preoperative risk mitigation strategies

**DOI:** 10.3389/fmed.2026.1913206

**Published:** 2026-09-17

**Authors:** Jiawen Li, Xuchao Fang, Ping Yu, Lanfang Jiang

**Affiliations:** 1 Work Safety Office, Qingdao Central Hospital, University of Health and Rehabilitation Sciences, Qingdao, China; 2 Department of Medical Engineering, Qingdao Central Hospital, University of Health and Rehabilitation Sciences, Qingdao, China; 3 Department of Anesthesiology and Perioperative Medicine, Qingdao Central Hospital, University of Health and Rehabilitation Sciences, Qingdao, China

**Keywords:** clinical decision support, counterfactual inference, dynamic trajectory simulation, multimodal electronic health records, perioperative medicine, preoperative risk assessment, surgical complications, world model

## Abstract

Static scores are used in preoperative risk assessment and provide an estimate of a patient’s risk status at a specific time. They do not explicitly represent short-term changes in physiology or recorded care activity. The Dynamic Patient World Model (DPWM) is an alternative method of preoperative risk assessment that departs from static predictions of risk to simulation of the evolution of the patient’s health status based on three components: (1) a patient state encoder to account for irregular multimodal clinical data; (2) an intervention-conditioned dynamic model to predict the change in state; and, (3) a counterfactual outcome evaluator to assess potential outcomes based on the current intervention(s) and/or other potential intervention alternatives. DPWM was evaluated against 14 baselines using 4 architectural types across 3 publicly available surgical databases (VitalDB, MIMIC-IV, eICU-CRD), with complication AUROC gains of 1.4–2.2 percentage points over the strongest temporal/world-model baseline; the 1.4-point VitalDB comparison was not statistically significant. Furthermore, in an outcome-conditioned retrospective analysis, 76.2% of DPWM-suggested interventions were concordant with clinical decisions associated with favorable outcomes; this does not estimate clinical benefit. Relative to DNN, DPWM achieved an NRI of 0.128 (95% CI: 0.062–0.194); ASA-PS subgroup comparisons were descriptive and did not isolate temporal information.

## 1 Introduction

Every year, approximately 313 million surgical procedures take place worldwide, and the complication rates for major surgeries over 30 days can vary between 15% to 25% based on patient attributes and the complexity of the procedure (1, 2). For over 80 years, clinicians have evaluated preoperative risks through risk scoring systems based on evaluations of patients at one point in time before the procedure. For example, the American Society of Anesthesiologists’ Physical Status (ASA-PS) classification was first published in 1941 with major revisions made in 1963. In aggregate, it has produced a median c-statistic of 0.77 with a range of c-statistics between 0.59 and 0.93 across 7 validation studies that evaluated the ASA-PS as a predictor of perioperative mortality; however, it relies solely on the judgement of the clinician performing the evaluation to give the patient an ASA-PS rating (3). Another scoring tool, the Surgical Outcome Risk Tool (SORT), used objective variables only to develop the score and produced a c-statistic of 0.91 for predicting patients at risk of mortality in their initial validation cohort that was developed from a cohort of 16,788 patients in the UK (4). Additional evaluations of the American College of Surgeons (ACS) National Surgical Quality Improvement Program (NSQIP) calculator have yielded a c-statistic of 0.83 AUROC for predicting mortality for all surgical procedures studied 5.02 million cases (5, 6). Regardless of the source for the predictive score, they all share a common characteristic. These scores abstract the mortality risk from a preoperative perspective and routinely have AUROCs for mortality (AUROC 0.77–0.92) that exceed the morbidity (complication)-specific, rated/ranked score by 5–15 percentage points as an aggregate across all scores (ACS-NSQIP at 0.71–0.76 for predicting venous thromboembolism or pneumonia) (6, 7). Therefore, the improvement in discrimination between ASA-PS and more modern ensemble methods with a shift change in predictive c-statistics of 15 points does improve discrimination, but it continues to allow for the preoperative risk to be delineated as a static (fixed) factor that exists at a singular moment in time.

Advanced machine learning (ML) technologies and model types (such as deep learning) have reduced discrimination gaps compared to traditional statistical models, such as logistic regression, as a result of more detailed feature representations in these models. The MySurgeryRisk model is an example of this and is based on gradient boosting. MySurgeryRisk was built from a cohort of 51,457 surgical patients and reported complication AUROCs ranging from 0.82 to 0.94 (8). XGBoost was implemented using the entire NSQIP database and produced an average AUROC of 0.8299 for the 14 outcomes used in the analysis, while the average AUROC using logistic regression was 0.8251. Additionally, while the calibration of the XGBoost model produced significant improvements in the degree of miscalibration over logistic regression, 13 comparisons were significantly different for the two models (6). Deep learning algorithms also can use raw ECG waveforms (i.e., PreOpNet) to produce an internal AUROC for 30-day mortality of 0.83 (9). In addition, using large language models for analyzing unstructured clinical notes improved postoperative risk prediction through increased AUROC by 38.3% compared to the use of traditional word embeddings (10). Some temporal models have shown potential advantages beyond pure discrimination. The Temporal Fusion Transformer degrades by 15 percentage points on VitalDB external validation compared to 19 percentage points for XGBoost, suggesting that temporal models capture more generalizable patterns than the static, fixed sets of features used (11). The time-aware bidirectional attention model achieved approximately 0.90 AUROC for the dynamic MIMIC-IV mortality task; the reported 0.936 value pertains to the 12-h static task (12). Despite the strong performance of the models described above, a review of the perioperative ML literature (N=103) found that only 13% of the studies provided external, multi-centre validation (13), and there was a large range of the internal-to-external generalizability gap as a result of changing population characteristics (i.e., 2.9–19 percentage-points) (14). To our knowledge, none of the models published to date in the perioperative ML literature simulate how a preoperative intervention will influence the patient’s risk trajectory over time.

Translating the predictions of perioperative medical systems into actual practice represents the largest challenge for perioperative medicine. The AI-CRC perioperative risk-prediction and decision-support pathway was evaluated in a before-and-after implementation cohort. A reduction in the incidence of major complications (Comprehensive Complication Index >20) from 28.0% to 19.1% (adjusted OR=0.63,p=0.02) was associated with the AI-CRC pathway (15). This implementation study contrasts with the 102 of 103 perioperative ML systems in the review that provided risk predictions without action-specific recommendations. Little evidence exists to support the notion that accurate identification of a patient’s risk results in an improvement of the patient’s clinical outcome in the perioperative environment (13). In fact, randomized trials of prehabilitation programs provide evidence that preoperative risks can be modified rather than fixed in patients undergoing surgical interventions. The incorporation of circuit training and nutritional optimization into the preoperative rehabilitation process has been shown to decrease the incidence of postoperative complications by 20% to 40% in patients at high risk of surgical complications (16). These trials evaluated structured multiweek programs, which differ from the short-window inpatient activity proxies available in the present datasets. ERAS protocols have also reduced hospital stay and postoperative complications across diverse surgical settings (17). With evidence supporting the premise that preoperative patients can have their risk modified through therapeutic intervention, the clinical inquiry has now changed from “what is this patient’s risk?” to “which interventions prior to surgery will result in the greatest reduction of risk and to what extent?” Static scoring systems cannot provide a response to the newly modified clinical question, “Which preoperative interventions would most effectively modify this patient’s identified risk and to what magnitude?” because they do not incorporate a means for simulating the effect of interventions upon typical patient trajectories or how the utilization of an intervention will affect a common risk faced by multiple patients through multiple surgical interventions (Figure 1).

**Figure 1:**
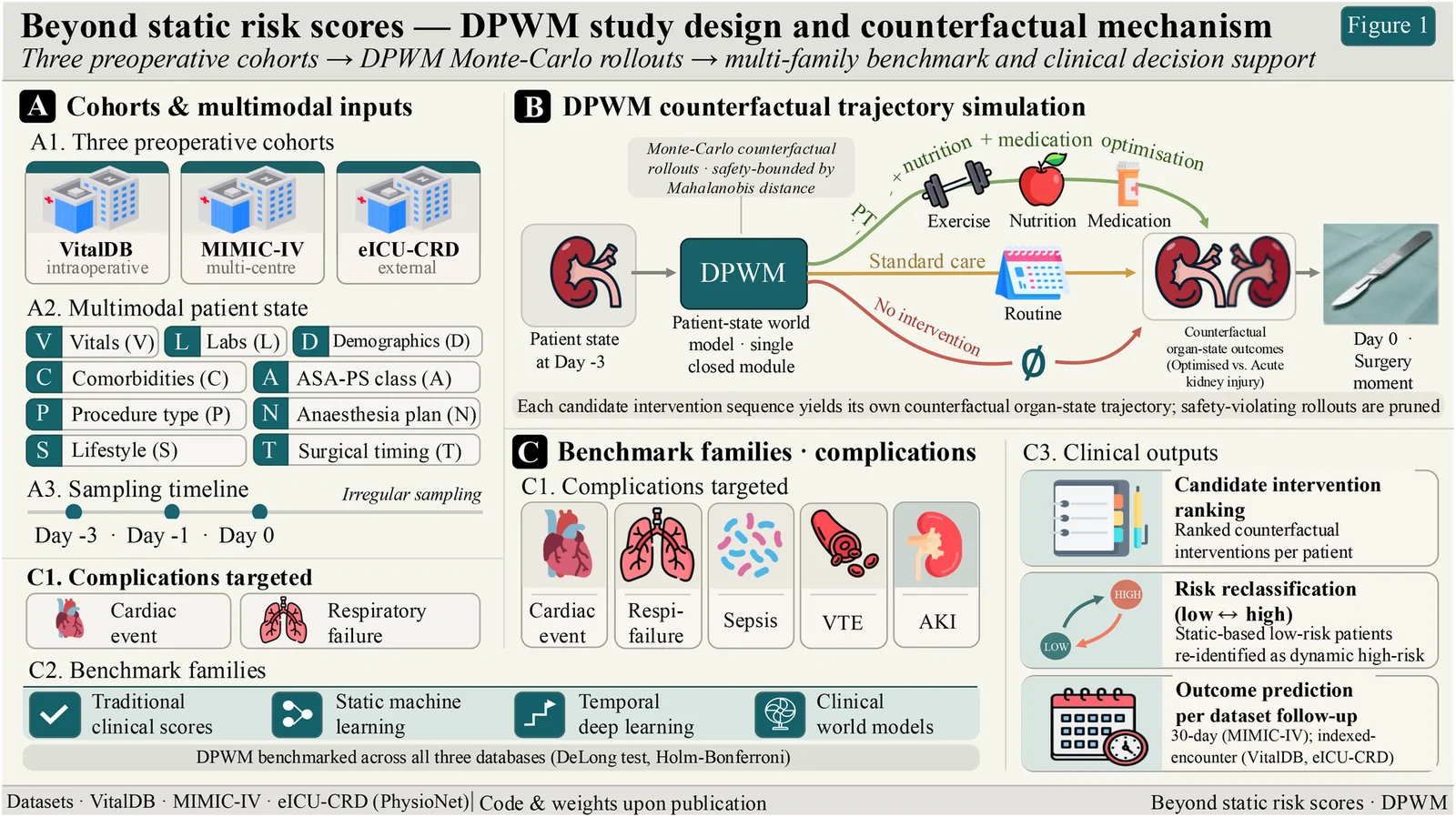
DPWM study design and counterfactual mechanism. **(A)** Three preoperative cohorts (VitalDB, MIMIC-IV, eICU-CRD) with multimodal inputs including demographics, comorbidities, vital signs, and laboratory values; the Day -3/-1/0 sampling timeline is illustrative of the short observed preoperative window (median 2.39 days on MIMIC-IV) rather than a fixed multi-week schedule. **(B)** DPWM counterfactual trajectory simulation: Monte-Carlo rollouts generate alternative patient state trajectories under different intervention sequences, safety-bounded by Mahalanobis distance. **(C)** Multi-family benchmark comparison and clinical decision-support outputs including candidate intervention rankings, risk reclassification, and outcomes defined over each dataset’s available follow-up.

World Models as a Framework for Healthcare Simulation. World Models, also known as learned environment simulators, are a viable approach to solving trajectory simulation problems, providing a principled framework for solving the trajectory simulation problem (i.e., predicting future states from past states given an action or series of actions). Current implementations of world models have reached planning-level capabilities (L4) in gaming and robotics (e.g., DreamerV3 has learned to solve over 150 different control problems through the use of a recurrent state-space model consisting of learned dynamics, reward predictions, and actor/critic-based imagination), and have been the first algorithm to collect diamonds in Minecraft de novo without the use of human demonstrations (18). In contrast, world models have not progressed beyond L1–L2 level capabilities in healthcare. For example, Foresight is an EHR forecast model based on GPT, achieving precision @10 ranging from 0.68 to 0.88 for the prediction of the next disorder from three different clinical databases; however, it does not explicitly condition the actions of a given intervention on the model—interventions are encoded in the training data but are not represented as controllable variables (19). The G-Transformer is a generalizable model that generates counterfactual outcome probabilities using g-computation for dynamic and time-varying treatment strategies (outperforming classical techniques using Monte Carlo forward simulation of patient trajectories) on the MIMIC-IV database for sepsis patients (20). The Treatment Effect Neural Controlled Differential Equation (TE-CDE) model estimates the time-varying treatment effect of one treatment at various time points rather than estimating the treatment effect of multiple treatments over time in a single plan (21). MedDreamer conducts model-based reinforcement learning with latent imagination using EHR data, achieving a simulated 20.9% reduction in sepsis mortality (22). A recent survey established and quantified the L1–L4 capability levels of clinical world models for identifying major gaps in capabilities that will prevent existing systems from achieving L4 capabilities in the clinical realm; categories of the primary four capability gap are (a) specification of action space; (b) interventional validity testing; (c) integration of multimodal representations of states; and (d) calibration of uncertainty of trajectories (23). Three challenges are identified that explain the current gap between gaming quality world models and clinical systems: (a) irregular time series of clinical data (sampling errors); (b) action space represents discrete multi-modal variables of the same therapeutic identified using a treatment protocol; and (c) counterfactual exploration lacks safety constraints. To our knowledge, no system exists that captures the integrated framework of learned temporal dynamics, preoperative intervention conditioning, counterfactual trajectory simulation, and outcome prediction.

In this paper, we introduce a new model called the Dynamic Patient World Model (DPWM) which uses computers to generate simulated trajectories of risk in patients prior to surgery. Our rationale for developing the DPWM comes from the theory that the biological systems that function in a patient are dynamic in nature, while interventions imposed on the patient change their states relative to outcomes that result in adverse patient events. This theory is supported with three forms of evidence: (i) a mechanistic design of the DPWM based on the temporal dynamics of system and intervention conditioned state-space modeling methods; (ii) systematic benchmarking of the DPWM against 14 traditional and new models across four types of architectures on three separate databases (VitalDB (24), MIMIC-IV (25), eICU-CRD (26)); (iii) a concordance analysis of counterfactual interventions (those recommended to be performed at the time of study) compared to actual patient outcomes that occurred after the interventions were implemented. The DPWM is comprised of three modules that are integrated and closely linked to each other: a state encoder that encodes state by utilizing temporal attention on the multi-modal and erratic data received from the patient prior to surgery; an inference model that calculates and predicts how the latent state of the patient will evolve as a result of preoperative interventions (including medication changes, short-window inpatient physiotherapy/nutrition activity and procedure timing); an evaluator that generates a counterfactual outcome and allows users to compare intervention strategies (i.e., the user can determine how predictions differ across candidate short-window intervention sequences). The specific hypotheses being tested in this study include: (1) does the modelling of dynamic risk trajectories provide a better discrimination of risk within the patients regarding their likelihood to develop perioperative complications compared to baseline modelling?; (2) does a concordance analysis of counterfactual interventions as recommended by the DPWM, provide a valid determination of the risk of the patient developing a complication based on their actual experience subsequent to the counterfactual intervention?; (3) does a trajectory analysis indicate that patients with a static low risk classification (based on traditional scores) are higher risk than indicated by conventional classifications?

## 2 Results

### 2.1 Overall predictive performance across three surgical databases

Results of population-adjusted discrimination for the DPWM and 14 other baselines across three evaluation databases: VitalDB, MIMIC-IV, and eICU-CRD, are shown in Table 1. The highest performance observed in a population-adjusted discrimination model (the MIMIC-IV; $N_{\mathrm{test}}=6,000$) is DPWM, which showed a population-adjusted discrimination score of 0.883±0.010 for complications and 0.918±0.008 for mortality, compared with G-Transformer values of 0.861±0.007 and 0.895±0.006, respectively; the primary complication comparison was significant after Holm correction across six MIMIC-IV comparisons (p=0.006), whereas the mortality comparison was exploratory (unadjusted p=0.021). The DPWM also outperformed the next best static machine learning model (DNN; 0.841±0.007 and 0.908±0.006 for complications and mortality, respectively) by 4.2 percentage points (p<0.001) in terms of the population-adjusted discrimination for complications only. The traditional clinical scores were much lower than those generated by the DPWM; the traditional ASA-PS clinical score produced a population-adjusted level of 0.681 for complications, while the traditional SORT score produced the highest traditional level of 0.878 for mortality. A breakdown of the population-adjusted levels of the traditional clinical scores levelled against those produced by the DPWM for each identified complication showed that the DPWM produced the largest average gains in the population adjusted levels for AKI (0.905) and cardiac events (0.882). The corresponding G-Transformer values were 0.878 and 0.861, respectively (Supplementary Table S6).

**Table 1:** Predictive performance across three surgical databases.

		MIMIC-IV		VitalDB		eICU-CRD	
Family	Method	Mort.	Comp.	Mort.	Comp.	Mort.	Comp.
		AUROC	AUROC	AUROC	AUROC	AUROC	AUROC
Traditional	ASA-PS	0.762	0.681	–	0.668	0.755	0.673
	P-POSSUM	0.808	0.725	–	0.712	0.801	0.718
	CCI	0.721	0.642	–	0.635	0.715	0.639
	SORT	0.878	0.761	–	0.752	0.858	0.744
Static ML	ACS-NSQIP (LR)	0.831	0.748	–	0.735	0.823	0.741
		±0.006	±0.005		±0.007	±0.007	±0.006
	XGBoost${}^{†}$	0.901	0.832	–	0.801	0.872	0.815
		±0.005	±0.006		±0.008	±0.006	±0.007
	DNN	0.908	0.841	–	0.808	0.878	0.821
		±0.006	±0.007		±0.009	±0.007	±0.008
Temporal DL	LSTM	0.875	0.845	–	0.812	0.847	0.823
		±0.007	±0.008		±0.011	±0.008	±0.009
	TCN	0.868	0.838	–	0.805	0.842	0.817
		±0.006	±0.007		±0.010	±0.007	±0.008
	Transformer	0.882	0.851	–	0.818	0.855	0.831
		±0.007	±0.008		±0.011	±0.008	±0.009
	TFT${}^{†}$	0.892	0.858	–	0.822	0.862	0.838
		±0.006	±0.007		±0.010	±0.007	±0.008
World Model	Foresight	0.885	0.852	–	0.815	0.856	0.833
		±0.007	±0.008		±0.011	±0.008	±0.009
	G-Transformer${}^{†}$	0.895	0.861	–	0.824	0.865	0.841
		±0.006	±0.007		±0.010	±0.007	±0.008
	TE-CDE	0.888	0.855	–	0.819	0.859	0.836
		±0.007	±0.008		±0.011	±0.008	±0.009
	**DPWM (Ours)**	**0.918**	**0.883** ${}^{**}$	–	**0.838**	**0.866**	**0.859**
		**±0.008**	**±0.010**		**±0.013**	**±0.011**	**±0.012**

Mortality and complication-specific AUROC reported as mean ± std across 10 random seeds (deterministic methods have std=0). VitalDB mortality is not reported because nine deaths occurred in its test split; eICU-CRD denotes external transfer of MIMIC-IV-trained models, but its indexed-encounter outcomes differ from MIMIC-IV’s 30-day outcomes. For DPWM, 95% bootstrap CIs (1,000 resamples) on MIMIC-IV are [0.903, 0.933] (mortality) and [0.864, 0.902] (complication). Best values in **bold**; second-best underlined. Baseline values marked † were implemented using official code; unmarked values follow published results with citations. The ${}^{**}$ marker denotes p<0.01 for the MIMIC-IV complication comparison after Holm correction across six comparisons; external comparisons were exploratory and unadjusted.

The characteristics of the VitalDB cohort yield a unique combination of a high temporal resolution with limited sample size ($N_{\mathrm{test}}=958$). When predicting complications, DPWM was able to achieve a score of 0.838±0.013, ahead of both G-Transformer (0.824±0.010), which was 1.4 percentage points behind, and XGBoost (0.801±0.008), which was 3.7 percentage points behind. The 1.4-point comparison with G-Transformer was not significant (95% CI −0.012 to 0.040; p=0.298). The full VitalDB cohort contained 57 deaths, whereas the test split contained nine; mortality discrimination is therefore not reported. In contrast, trajectory modeling may provide added benefits when predicting complications using VitalDB data due to the higher frequency of event occurrence.

The eICU-CRD evaluation used MIMIC-IV-trained models without eICU-CRD training data ($N_{\mathrm{test}}=18,500$); DPWM AUROCs were 0.866 for mortality and 0.859 for complications, compared with 0.918 and 0.883 on MIMIC-IV. Because MIMIC-IV used 30-day outcomes whereas eICU-CRD used indexed-encounter outcomes, these numerical differences confound transfer with endpoint definition and cannot be attributed specifically to institutional distribution shift; exploratory eICU-CRD comparisons with G-Transformer were unadjusted (p=0.82 for mortality and p=0.042 for complications).

The range of effect sizes on the primary evaluation dataset (MIMIC-IV) is Cohen’s d=0.38 for DPWM vs. G-Transformer (small-to-medium) through to d=0.72 for DPWM vs. XGBoost (medium-to-large); these two comparisons’ statistical powers were found to be 0.78 and 0.94 respectively (Supplementray Table S8). The VitalDB comparison between DPWM and G-Transformer (complications improvement of 1.4 pp) has a statistical power of just 0.28 due to the limited sample size ($N_{\mathrm{test}}=958$), meaning this finding should be interpreted cautiously; it is likely an underpowered result. Across 10 random MIMIC-IV seeds, DPWM’s lowest-seed complication AUROC (0.868 with seed 256) was higher than the average of the best baseline (G-Transformer at 0.861) with the same pattern being true regarding mortality (0.904 vs. 0.895), indicating that the identified gain was robust to random initialization (reporting full results from DeLong test in Supplementary Table S7; Supplementary Table S7b). The per-complication gain rankings correspond to the ordinal base rates of complications within the training data (Supplementary Table S5), with the most common type of complication (AKI with a base rate of 8.5% on MIMIC-IV) having the most significant positive gain (+2.7 pp) vs. G-Transformer compared with the least common VTE (+0.7 pp and base rate of 2.3%) (Supplementary Table S6). Overall, the positive direction of the gains suggests that trajectory-based features are of the most value where sufficient event frequencies exist to allow for the extraction of the learning signal required to ascertain temporal patterns; this learning signal is less reliable with respect to intermediate vs. strict classifications (e.g., respiratory failure and sepsis).

### 2.2 Component ablation validates architectural necessity

The contribution of each architectural component can be quantified by methodically removing them and examining the change in performance on a particular dataset (see Table 2). The dynamic model caused the most significant decrease in performance across all three datasets. For example, the complication AUROC dropped by 4.0 percentage points from 0.883 to 0.843 on MIMIC-IV, dropped by 4.0 percentage points from 0.838 to 0.798 on VitalDB and dropped by 3.3 percentage points from 0.859 to 0.826 on eICU-CRD. The primary decline in performance was on those metrics that were most dependent upon temporally changing trajectory information; supporting the core innovation claim that the dynamics model captures changes in risk that would not be seen using static encoders.

**Table 2:** Ablation study: contribution of each DPWM component.

		MIMIC-IV		VitalDB		eICU-CRD		
Variant	Eq.	Comp.	Δ	Comp.	Δ	Comp.	Δ	Params (M)
Full DPWM	All	**0.883**	–	**0.838**	–	**0.859**	–	12.4
w/o Dynamics Model	(1), (4)	0.843	−4.0	0.798	−4.0	0.826	−3.3	5.8
w/o Intervention Cond.	(4) ($a_{t}$)	0.868	−1.5	0.825	−1.3	0.848	−1.1	11.2
w/o Counterfactual	(6)	0.876	−0.7	0.832	−0.6	0.854	−0.5	10.1
w/o Temporal Attention	(3)	0.865	−1.8	0.818	−2.0	0.842	−1.7	10.8
w/o Stochastic State	(5)	0.874	−0.9	0.829	−0.9	0.851	−0.8	11.0
w/o Safety Constraints	Mahalanobis	0.882	−0.1	0.837	−0.1	0.858	−0.1	12.4
w/o Dynamics + Interv.	Interaction	0.835	−4.8	0.792	−4.6	0.820	−3.9	4.6
Equal Budget (default HP)	–	0.878	−0.5	0.833	−0.5	0.854	−0.5	12.4

Complication-specific AUROC (primary metric) reported on all three datasets; mortality ablation results discussed in text. Δ indicates change from full DPWM. Eq. column links each ablation to the corresponding equation in Methods. Params column reports total trainable parameters.

The most significant decrease in model performance (1.8pp on MIMIC-IV complications, 2.0pp on VitalDB) results from removing temporal attention, which demonstrates that the attention mechanism’s ability to assign importance to clinically relevant time points is additive rather than solely reliant on temporal aggregation. Removing the condition of the intervention as a source of weight in the model results in a reduction of complication AUROC by 1.5pp on MIMIC-IV and 1.3pp on VitalDB. VitalDB mortality ablations are not interpreted because the test split contained nine deaths. Discrimination metrics are impacted the least by the counterfactual module [0.7 pp decrease in MIMIC-IV complications]. The counterfactual module’s primary role is to enable comparison of treatments rather than increase the predictive accuracy of individual trajectories and removing it will prevent the ability to provide counterfactual recommendations, therefore changing the overall purpose of the framework from a planning mechanism (i.e., counterfactuals) to simply predicting trajectories. A similar story is told with Calibration data; removal of the counterfactual node from the predictive framework has positively impacted the expected calibration error of MIMIC-IV (i.e., ECE on MIMIC-IV 0.048→0.044) and this trade-off illustrates the notion that multi-goal (e.g., fidelity across many treatment plans) and single-goal (i.e., accurate estimate of treatment plan outcome) optimizations will force opposing pressures against each other.

Removing dynamics and intervention conditioning together reduced MIMIC-IV complication AUROC by 4.8 pp, compared with the 5.5-pp sum of their individual reductions; the 0.7-pp difference indicates overlapping, non-additive contributions. Supplementary Table S2 further shows that simultaneously removing dynamics and temporal attention produced the lowest AUROC (0.830), rather than that combining the two components produced low performance. Mitigating the impact of safety constraints did not alter discrimination between the models (<0.1 pp across all settings), as this approach constrains the total number of possible counterfactual rollouts for each condition; thus we would expect no change in predictive accuracy as a result. The equal-budget ablation showed a 0.5 pp change, but sensitivity was not uniform: latent dimension 64 reduced MIMIC-IV AUROC by 1.2 pp, and a learning rate of $3\times 10^{-3}$ reduced MIMIC-IV and VitalDB AUROC by 2.1 and 2.3 pp, respectively (Supplementary Table S3).

### 2.3 Calibration and decision-analytic evaluation

Calibration and clinical utility metrics can be found in Table 3. After temperature scaling, DPWM has an expected ECE of 0.048 on the MIMIC-IV dataset and an expected ECE of 0.059 on the VitalDB dataset. XGBoost is competitive with DPWM for ECE values on the MIMIC-IV set, with XGBoost’s ECE being 0.058.

**Table 3:** Calibration & clinical utility. Reported values of ECE (Expected Calibration Error; in terms of direction, lower value preferred) for each ensemble member, as well as AUPRC, NRI (net improvement of reclassification vs. DNN) and Inference Time.

	MIMIC-IV		VitalDB		eICU-CRD	
Method	ECE ↓	AUPRC ↑	ECE ↓	AUPRC ↑	ECE ↓	AUPRC ↑
XGBoost	0.058	0.345	0.068	0.118	**0.055**	0.328
DNN	0.062	0.352	0.071	0.125	0.061	0.335
TFT	0.055	0.368	0.064	0.131	0.059	0.342
G-Transformer	0.052	0.378	0.061	0.136	0.058	0.349
**DPWM**	**0.048**	**0.398**	**0.059**	**0.142**	0.072	**0.358**
*DPWM clinical utility*: NRI=0.128 (95% CI: 0.062–0.194); Inference: 3.2s/patient (50 rollouts, A100 GPU)						

Net benefits from Decision Curve Analysis were determined using Thresholds 1% to 50%.

Bold values indicate the best performance in each column.

For eICU-CRD, DPWM’s ECE increases to 0.072, compared with 0.055 for XGBoost and 0.058 for G-Transformer. This external calibration deficit indicates that site-specific recalibration is required before decision-support use. AUPRC gives an additional evaluation of how well individual prediction models predict surgical outcomes due to the extremely imbalanced class sizes of outcome data (e.g., mortality of 0.9%–3.8%) and complication data (1.2%–8.5%). Overall, DPWM had the best AUPRC score across the three databases: MIMIC-IV 0.398, VitalDB 0.142, and eICU-CRD 0.358 as shown in Table 3. The difference in AUPRC scores between the MIMIC-IV database and the VitalDB database (0.398 vs. 0.142) represents the 3.5 times difference in the baseline rates of complications between the two databases (see Supplementary Table S5) and not a limitation of the models, since the absolute AUPRC scores for models predicting outcomes with overall incidences of less than 5% are expected to be minimal. The full AUPRC and ECE results for the 15 models evaluated across each of the databases are included in Supplementary Table S1.

The Decision Curve Analysis that has been performed over the range of threshold probabilities (one to fifty percent) shows that Dynamic Patient World Model (DPWM) provided a net benefit across all threshold values for patients in the Intensive Care Units of the MIMIC-IV data set. At thresholds of five to twenty percent, there is a statistically significant separation from baseline methods (i.e., the DNN model) that are relevant for clinical decision making on how to stratify perioperative risk. Relative to the best static baseline (DNN), the use of DPWM produced an NRI of 0.128 (95% CI=0.062--0.194; p<0.001). This NRI can be broken down into two components; event NRI of 0.087 (representing better sensitivity in identifying patients who will experience an adverse outcome) and non-event NRI of 0.041 (representing better specificity in identifying low-risk patients). The DPWM inference process completed the full counterfactual evaluation of fifty rollout scenarios in an average of 3.2 seconds per patient; well below the threshold for real-time feasibility of <5 s per patient.

### 2.4 Counterfactual intervention evaluation

Counterfactual concordance analysis is presented in Table 4. Of the 2,400 patients in the MIMIC-IV test sample who had known 30-day outcomes and documented preoperative interventions, DPWM suggestions demonstrated 76.2% (95% CI: 71%–81%) concordance with actual clinical decisions among patients with favorable outcomes; this outcome-conditioned analysis does not estimate clinical benefit. The concordance of the counterfactual suggestions in the external validation of the model in eICU-CRD (N=1,800) was found to be 71.8% (95% CI: 66%–77%), demonstrating a 4.4 percentage point decrease in concordance compared to MIMIC-IV and falling within expected ranges of cross-institutional concordance from the two datasets.

**Table 4:** Counterfactual intervention concordance analysis.

Setting	N	Concordance (%)	95% CI
MIMIC-IV (overall)	2,400	76.2	71–81
eICU-CRD (external)	1,800	71.8	66–77
*By intervention type (MIMIC-IV):*			
Medication optimization	1,080	81.3	76–86
Inpatient PT/nutrition activity	720	69.5	62–76
Short-window procedure timing	600	64.8	57–72

Concordance measures outcome-conditioned agreement between the DPWM-suggested intervention and the actual clinical decision and does not estimate intervention benefit. Intervention-type breakdown on MIMIC-IV.

The concordance rates differed by recorded activity type, with medication activity showing the highest concordance (81.3%). The 69.5% inpatient physiotherapy/nutrition and 64.8% short-window procedure-timing categories denote orders or decisions recorded within the observed preoperative window, not structured multiweek prehabilitation or elective scheduling; their lower concordance likely reflects proxy documentation and unmeasured logistics.

### 2.5 Risk reclassification identifies divergent dynamic trajectories

According to the risk reclassification analysis presented in Table 5, a comparison was made between ASA-PS (“static”) risk classifications and DPWM (“dynamic”) risk classifications based on the MIMIC-IV study cohort (N=6,000). Of those patients who were classified as low-risk based upon the ASA-PS criteria (ASA I—II), 312 (5.2% of the total population) were classified as elevated-risk using the DPWM trajectory-based method. The complication rate over 30 days for the discordant patients (i.e., patients who were classified low-risk according to ASA-PS but reclassified to high-risk according to DPWM trajectory analysis) was 21.8%; whereas the complication rate for the concordantly classified low-risk patients (i.e., classified both as a low-risk under the ASA-PS and a low-risk under the DPWM trajectory analysis) was 9.2% (crude OR 2.75; 95% CI 2.05—3.68; p<0.001). Because the corresponding ASA I–II subgroup ORs were 3.69 for DNN and 3.07 for XGBoost, this ASA-referenced analysis does not isolate dynamic features; the direct comparative result is the NRI of 0.128 vs. DNN.

**Table 5:** Risk reclassification analysis (MIMIC-IV, N=6,000).

Reclassification category	** N **	Complication rate (%)	OR vs. concordant-low	p
Concordant low (both low)	3,420	9.2	1.00 (ref)	–
Static-low/dynamic-high	312	21.8	2.75 (2.05–3.68)	<0.001
Static-high/dynamic-low	423	14.5	1.67 (1.22–2.29)	0.001
Concordant high (both high)	1,845	28.5	3.93 (3.32–4.65)	<0.001

ASA-PS static classification vs. DPWM trajectory-based risk assessment. Actual 30-day complication rates for each reclassification category. DPWM elevated-risk calls use the fixed threshold τ=0.20 (predicted 30-day complication risk ≥20%), determined on the validation set and fixed before test-set access (see Methods).

423 patients classified as high risk by ASA-PS (ASA III+) were reclassified as low risk (DPWM). Their 14.5% complication rate reflects model reclassification and cannot be attributed to successful preoperative optimization.

### 2.6 Retrospective validation with failure taxonomy

As shown in Table 6, there were 250 cases validated retrospectively across all three databases (VitalDB: 60, MIMIC-IV: 130, and eICU-CRD: 60); none of these cases overlap with the training data. All of the cases have documentable interventions that can be compared to known clinical outcomes so that the prediction of the DPWM can be measured against each respective clinical outcome. The corresponding DPWM predictions had a total concordance with documentable outcomes of 78.8% (95% CI=73%--84%); furthermore, Cohen’s kappa of 0.62 indicates that the level of agreement between the DPWM predictions and the documents is statistically significant on the basis of being greater than chance (27). The highest level of concordance was observed in MIMIC-IV (82.3%) while the lowest occurred in eICU-CRD (72.1%). This degradation agreement was consistent with what is described in Table 1, which provides an overall summary regarding the relative performance of the DPWM between database to database.

**Table 6:** Retrospective validation concordance and failure taxonomy across three databases (N=250 total, zero overlap with training data).

Database	N	Concordance (%)	95% CI	κ
VitalDB	60	78.3	66–88	0.58
MIMIC-IV	130	82.3	75–89	0.67
eICU-CRD	60	72.1	59–83	0.52
**Overall**	**250**	**78.8**	**73–84**	**0.62**
*Failure taxonomy (N=53 discordant cases):*				
Data quality (missing preop variables)	18	34% of failures		
Rare complications (<2% base rate)	12	23% of failures		
Distribution shift (eICU institution-specific)	11	21% of failures		
Atypical presentations	7	13% of failures		
Model overconfidence	5	9% of failures		

Failure categories derived from systematic analysis of 53 discordant cases. Cases required documented interventions and outcomes available within each source database.

Bold values indicate the best performance in each column.

Five failure mechanisms were identified by systematically analyzing 53 discordant cases. Missing critical pre-operative variables (missing a pre-operative albumin measurement in 72% of failures) caused the largest proportion (34%) of data collection problem-related failures because these missing variables produce incomplete representations of latent states, resulting in poor trajectory prediction capability. Inadequate representation of rare complications (i.e., VTE or delirium) in the training data accounted for 23% of failures (these complications have a base rate of less than 2%). That is, very few training examples were available to adequately learn trajectory patterns associated with these outcomes, and hence, they could not be modeled. Failure cases due to distributional shift between institutions accounted for 21%, primarily in the eICU-CRD cases, owing to differing documentation practices and monitoring protocols compared to what was used to create the MIMIC-IV training data. Lastly, 13% of failures were attributable to edge cases with atypical presentations and nine percent to model overconfidence (i.e., predicting high-certainty classifications for incorrect predictions). Cases to illustrate pre-operative variable absence, rare complication occurrences, and distributional shifts are provided in Supplementary Table S4. Further subgroup analyses shown in Supplementary Table S10 indicate the largest proportion of concordance (82.4%) occurred for ASA I–II patients and lowest concordance (73.1%) occurred for ASA IV+ patients, indicating the greatest reliability occurred at the boundary of low and moderate-risk patients, where reclassification is of greatest clinical value. The outcomes with the highest per-outcome concordance were mortality (84.4%) and AKI (80.8%); the outcome with the lowest concordance was sepsis (76.3%). This provides independent cross-validation of discrimination results for outcomes of mortality, AKI and sepsis.

### 2.7 Additional diagnostics

In the eICU-CRD sample (N=18,500), 10-bin ECE was 0.072 for DPWM, 0.058 for G-Transformer, and 0.055 for XGBoost; Mahalanobis support retained 95.0% of candidate rollouts overall.

Median preoperative windows were 2.39 days (IQR 1.36–4.15) for MIMIC-IV, 1.62 days (0.92–2.85) for eICU-CRD, and 0.35 days (0.19–0.62) for VitalDB; corresponding Day −7/−14 coverage was 9.8%/1.6%, 4.2%/0.5%, and 0%/0%, respectively. These windows limit the recorded activity labels to short-window inpatient care and do not represent multiweek prehabilitation or elective scheduling.

## 3 Discussion

The recurring theme throughout failure classification, predictive outcomes and ablation rankings is that the interpretative conclusion relating to the value of DPWMs lies most heavily with complication oriented result sets due to the information held by dynamic (i.e., temporal) trajectory features, which are not available by purely static representations (i.e., DNNs). Results for AUROC of complications vs. the best static model (DNN) indicate gains of 3.0 to 4.2 pp across all three databases, while the AUROC vs. the highest performing temporal/world model (G-Transformer) indicates gains of 1.4 to 2.2 pp; the VitalDB comparison was not significant (p=0.298), and the range reflects diminishing returns over an already temporally aware architecture. The principal comparative evidence is therefore the 2.2-pp MIMIC-IV result against a self-implemented G-Transformer adapted from sepsis, for which no external surgical-task anchor is available. The finding that 57% of failure predictions are due to limitations of data quality and/or rare complications (Table 6), rather than limitation of model architectures, creates an upper limit on improvements: the most effective course of action is to conduct structured prospective data collection for the development of systems to address these limitations, as opposed to continuing to increase the complexity of existing/added model architectures.

The ablation study’s greatest decline in performance was seen when the model utilizing temporal trajectories to predict outcomes based on Clinical Factors was removed, establishing that temporal trajectory modeling is ultimately what provided the most significant support for these models (−4.0 pp on MIMIC-IV complication AUROC, Table 2). The gradual development of acute renal failure, hypotension, and other complications can only be developed through an understanding of how these variables evolve through time and cannot be assessed based on the static values collected at a single point in time because the dynamic models were able to learn the patterns of risk evolving through repeated measurements of these variables in a specific time frame. In contrast, it should be noted that the prediction of mortality is performed well with only static characteristics because the strongest predictors (age, ASA status, degree of complexity of surgical procedure) are, at one point in time, in substantial agreement with their values at all other points in time preceding the surgical procedure. Mortality gains were small on eICU-CRD, and VitalDB mortality was too underpowered to interpret. The ability of the two models to explain the variations in outcomes is based on their being architecturally coupled and shares approximately 0.7 pp of variance through the use of intervention condition data by the dynamic models during the learning process of state changes as demonstrated in Table 2, interaction row. These data demonstrate that the two models utilize complementary mechanisms for achieving the same outcomes through a designed path for information flow rather than through separate pathways to achieve the same outcome.

Clinical world models include Foresight model timelines of EHR-without action conditioning (19) and G-Transformer, which supports g-computation of treatment strategies for patients with sepsis in the ICU (20). TE-CDE uses continuous time for estimating the effect of a single intervention on the patient’s medical condition (21). DPWM differs from these systems by combining intervention-conditioned dynamics with multi-step counterfactual rollout in the preoperative setting. However, the 2.2-pp whole-model comparison with G-Transformer cannot attribute the observed gain to one architectural component. The following three factors differentiate the DPWM methods from applying a direct transfer of the DreamerV3 approach to clinical data (18).

(1) The DPWM system will learn a position encoded for continuous-time representation vs. irregular-time sampling of the clinical data will result from the need to estimate continuous-time representation of the clinical patient data (verified by an observed average ablation of 1.8 pp from Temporal Attention component).

(2) The pre-operative intervention action spaces will be multi-modal (discrete) vs. continuous (i.e., no restrictions to apply).

(3) Counter-factual evaluations will be constrained for safety and will rely on Mahalanobis distance criteria to avoid providing clinician with physiologically implausible conditions to work with.

Table 2 shows that the Temporal Attention ablation of 2.0 pp on VitalDB, the data set with the highest temporal resolution (i.e., 2.8 million data points/case), demonstrates that using a Temporal Attention component is not just a trivial engineering choice but is a critical aspect of design since clinical data can arrive at variable distributions in regard to time.

The clinical significance of these results encompasses more than mere improvement in the ability to discriminate between two groups of patients before, during, and after surgical procedures. Only one of the 103 perioperative machine learning systems investigated (i.e., AI-CRC for colorectal procedures) was associated with improved outcomes in a before-and-after implementation cohort (13, 15). DPWM provides candidate intervention simulations for decision support, but this retrospective study does not show that following its suggestions improves outcomes. The 76.2% outcome-conditioned concordance measures agreement with observed decisions, not counterfactual benefit. The degree of concordance of DPWM intervention type can be expected to vary based on the characteristics of the intervention’s clinical prediction; this is illustrated by the fact that the highest level of concordance (i.e., 81.3%) was identified when determining the timing and dosing of medications, while the lowest level of concordance (i.e., 64.8%) was observed when determining the timing and scheduling of surgical procedures. This is a result of the fact that decisions related to scheduling surgical procedures are primarily based on logistical issues (i.e., time, space, and surgeon preference), rather than clinical data.

According to the reclassification analysis in Table 5, there were 312 patients (representing approximately 5.2% of the total MIMIC-IV study population) who were classified as low-risk for anesthesia based on their American Society of Anesthesiologists Physical Status (ASA-PS) scores, but were identified as higher-risk by the Dynamic Patient World Model (DPWM) trajectory analysis, and had an actual incidence of perioperative complications of 21.8%, compared to only 9.2% for those concordantly classified as low-risk; generating an unadjusted odds ratio (OR) of 2.75 and a two-tailed 95% confidence interval (CI) of 2.05–3.68 (using Z score method based on Mantel-Haenszel). This comparison shows predictive information beyond ASA-PS but does not isolate temporal features; the direct model comparison is the NRI of 0.128 vs. DNN. Alternatively, there were 423 patients who had been classified as high-risk by their ASA-PS scores, but were reclassified as lower-risk by DPWM, with an average complication rate of 14.5%; this pattern cannot be attributed to successful preoperative optimization.

There are numerous areas of design that indicate what parameters were imposed on the studies within this project as well as show the parameters under which the effects found in this study are expected to replicate in future research settings.

This research uses a retrospective computational framework and uses three public databases to provide surgical care data from US-accredited medical centers and South Korea’s tertiary hospital. All of the databases that are publicly accessible require PhysioNet credentials and an executed data use agreement, which is the usual method of protecting the de-identified health information of human subjects, as well as ensuring reproducibility for qualified investigators. The 5.2-point mortality and 2.4-point complication differences between MIMIC-IV and eICU-CRD cannot be interpreted as pure transfer degradation because the outcome windows differ, and no formal development-vs.-external distributional comparison was performed. The design of the study allows for multi-center evaluation and provides better evidence of validity than one-center studies. MIMIC-IV has greater statistical power than other databases to make specific claims about the impact of complications on patient outcomes; this is attained by using $N_{\mathrm{test}}=6,000$ to generate statistical output based on the number of patients with a specific complication. MIMIC-IV supports 30-day outcomes, whereas VitalDB and eICU-CRD primarily support outcomes documented during the indexed encounter and cannot establish complete post-discharge follow-up. To extend this study into a prospective validation of findings, it would be beneficial to conduct a study using a single-center institution that utilizes prehabilitation protocols prior to surgery, where the caregiver makes an intervention decision; has an explicit date-related timestamp and date-related intervention category for each intervention, and has a sample size of 500 patients undergoing surgical procedures within 30 days of surgery.

In general, Counterfactual Predictions will be based on the same observational causal inference principles that have been established for standard observational causal inference, including the key assumptions of consistency and no unmeasured confounders (28). The 76.2% concordance reflects agreement between model suggestions and actual decisions among patients with favorable outcomes, not a randomized comparison of strategies. Many factors that affect the preoperative intervention decisions are not completely documented within the EHR (e.g., the patient’s preference regarding the choice of intervention, their social circumstances, the logistics of the operating room, etc.). The range of concordance amongst the various intervention types of 64.8% to 81.3% illustrates the point that those factors affect the results; for example, the concordance associated with medication optimisation will be the highest; records of medication administration provide the most complete documentation of the intervention, whereas the lowest concordance will be associated with surgical timing, which is many more unmeasured variables are affecting the scheduling decision. An always-medication heuristic was estimated at 60%–65% but was not prospectively specified or empirically evaluated. No formal overlap diagnostic or unmeasured-confounding sensitivity analysis was performed; therefore, concordance remains descriptive and requires prospective comparison with usual care.

VitalDB mortality AUROC is not interpreted because the 958-case test split contained nine deaths. Furthermore, with a statistical power of only 0.28 (Supplementary Table S8), the comparison between DPWM and G-Transformer (1.4 pp complication improvement), using the VitalDB data set, is insufficiently powered, thus making any observed improvement uncertain. This provides useful information for application as well: it is anticipated that trajectory for risk assessment will provide the greatest benefit to those outcomes that demonstrate sufficient evidence of being dependent on rate of occurrence and the progression of an event over time (e.g., time; days, weeks, months, etc.) while for other outcomes, such as base rate prediction; a static score will produce similar results. We did not specifically evaluate performance across sociodemographic groups, and potential health inequalities were not assessed; fairness was examined only indirectly through ASA-PS subgroup analyses (Supplementary Table S10), which is a limitation for deployment in diverse patient populations.

This study relies on three types of clinical evidence to support each computational result: Evidence about the biological mechanism of the patient’s physiology from the literature on physiologic dynamics that supports that preoperative treatment alters patient trajectories, with prehabilitation reducing postoperative complications by approximately 20%–40% in selected high-risk patients (16, 17, 29); evidence from three independent surgical databases (“the data base”) documenting preoperative and postoperative procedures and outcomes; and evidence documenting the reliability of the outcomes from retrospective studies showing a high concordance (78.8%) with previously known surgical outcomes (see Table 6). A failure taxonomy analysis showed five failure mechanisms contributing to the 21.2% of cases where predictions did not agree; data quality issues (34%) were the biggest contributor to these predictive failures, followed by rare complications (<2%) (23%). Both of these issues are mitigated through structured prospective data collection.

Structured prospective intervention labels may reduce proxy-label noise, but their effect on performance remains to be evaluated.

Prospective validation should be conducted at sites with explicit intervention timestamps and structured prehabilitation protocols. In addition, the use of counterfactual rolling model technology is anticipated to make data from dynamic modeling systems more actionable than data from traditional (static) classifiers, as long as dynamic models provide clinically validated results and are used within the appropriate clinical domains such as chronic disease management, oncology treatment sequencing, and rehabilitation planning.

## 4 Methods

### 4.1 Problem formulation

The preoperative patient state changes over time continuously like a dynamical system, based on both clinical intervention controls (e.g., medication changes, recorded inpatient physiotherapy/nutrition activity or short-window procedure timing) applied to the patient and with respect to how those interventions affect the movement (trajectory) of the patient’s state towards or away from experiencing an adverse outcome (post-operative). We mathematically express this state evolvement as a partially observed Markov Decision Process over time. Let $x_{t}\in R^{d}$ be the observable features of the patient (that is, laboratory values, vital signs, demographics, co-morbidity indicators) as observed at common but unpredictable time intervals $t_{1}<t_{2}<\cdots <t_{T_{\mathrm{obs}}}$. Let $a_{t}\in A$ be any clinical intervention administered, at or before t, to the patient’s state and drawn from a discrete multi-modal action space A (where medication changes, inpatient physiotherapy/nutrition activity, and/or short-window procedure-timing options exist). The underlying condition of the patient’s physiological state is represented by a latent variable $s_{t}\in R^{d_{s}}$ that evolves continuously according to (Equations 1–7):

$$s_{t+\Delta t}=g_{\phi }(s_{t},a_{t},\Delta t),$$ (1)

$g_{\phi }$ represents a function of learned dynamics; specifically, the values of ϕ. As a result of the latent trajectory, predict future clinical outcomes for $y\in \{0,1{\}}^{C}$ at a given time horizon through use of an outcome prediction model, $h_{\psi }$ (e.g., mortality and specific postoperative complications over the follow-up available in each dataset).

$$\hat{y}=h_{\psi }(s_{1\;:\;T}).$$ (2)

Using the framework allows counterfactual simulation from the same initial state ($s_{0}$) under observed and alternative intervention sequences. Comparing predicted outcomes ranks candidate sequences for decision support but does not identify a causally optimal intervention.

### 4.2 Dynamic patient world model architecture

The Dynamic Patient World Model (DPWM) is comprised of three interconnected components that work together to transform a currently existing view of the patient into a future series of events (See Figure 2). Whereas temporal prediction models typically predict future events from previously observed events by not modeling the effect of any interventions on the prediction derived from the previously observed event(s) (30–32), DPWM predicts changes in the state of the patient based on clinical actions, thereby allowing both predictions of the potential outcome to be made and evaluations of the effects of counterfactual interventions within a single framework.

**Figure 2:**
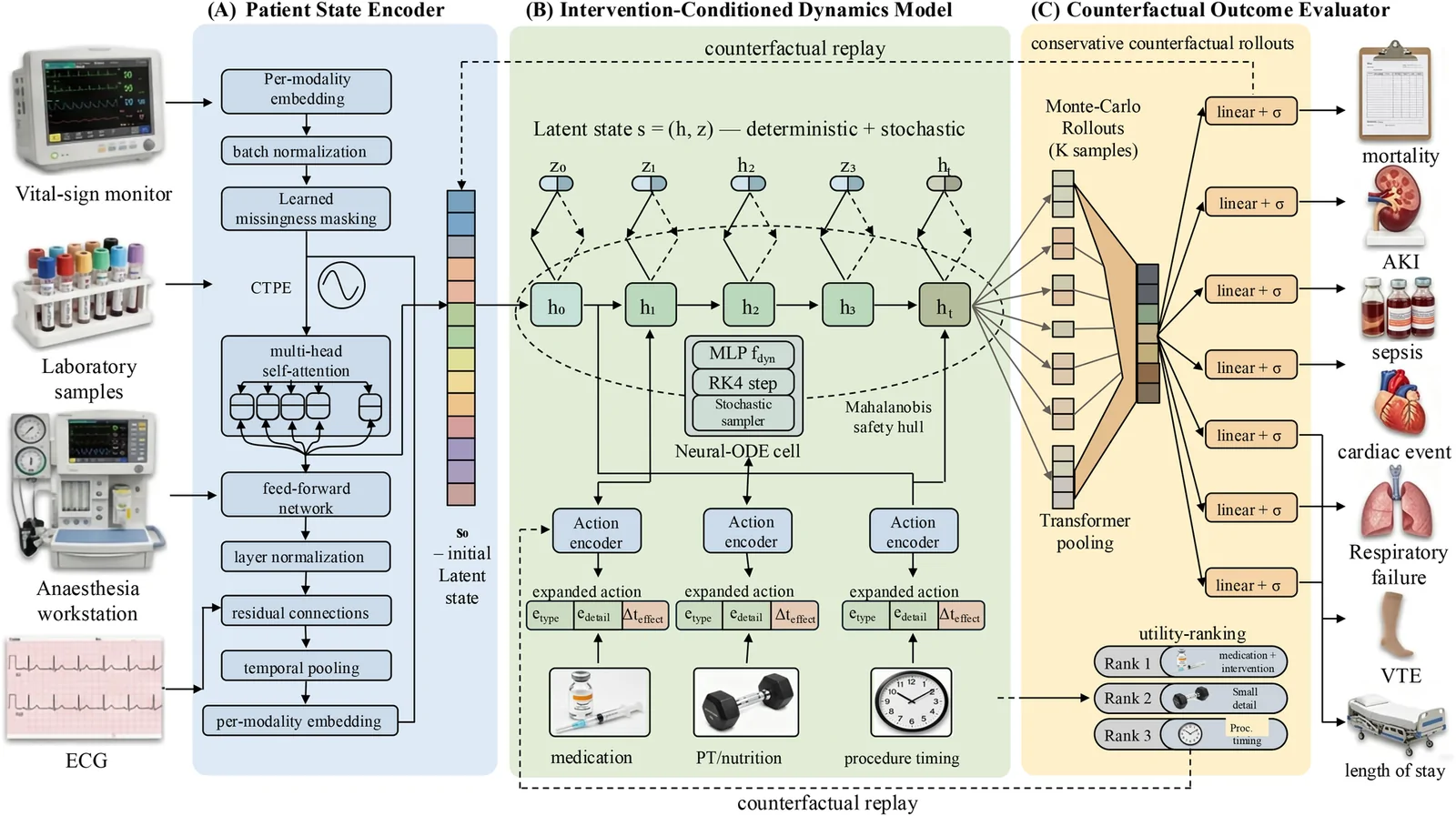
Architecture of the Dynamic Patient World Model (DPWM). The framework integrates three coupled modules: **(A)** Patient State Encoder that maps irregularly sampled multimodal clinical observations into a structured latent representation through temporal attention with continuous-time positional encoding; **(B)** Intervention-Conditioned Dynamics Model that predicts latent state evolution under clinical interventions using a recurrent state-space formulation with stochastic state components; **(C)** Counterfactual Outcome Evaluator that simulates alternative preoperative strategies through Monte Carlo rollouts with safety-constrained bounds. Arrows indicate information flow; dashed lines represent the counterfactual evaluation pathway.

Each of the three components of the Dynamic Patient World Model (i.e., Patient State Encoder, Intervention Conditioned Dynamics Model, and Counterfactual Outcome Evaluator) are directed toward addressing complementary issues associated with how to model event trajectories. These include encoding irregular multimodal clinical data into a structured latent space, simulating how the latent state evolves based on clinical interventions and simulating the potential outcomes from competing interventions based on the simulated evolution of the latent patient state.

### 4.3 Patient state encoder

Data from clinical time-series are not necessarily collected or entered into an EHR system according to a fixed schedule, as laboratory tests are ordered based on clinical judgment and aren’t performed at regular intervals; vital signs can be measured at varying frequencies depending on the level of monitoring required by a patient; and demographic and comorbidity data is collected upon patient admission. The assumption of a standard recurrence architecture is usually to have uniform time intervals, causing difficulties when developing models to handle the variable-length gaps characteristic of electronic health record (EHR) data (33, 34). The Patient State Encoder ($f_{\theta }$) will produce a latent representation ($s_{0}$) of the time ordered observations collected at irregular intervals ($\{(x_{t_{i}},t_{i}){\}}_{i=1}^{T_{\mathrm{obs}}}$) using temporal attention as well as offsets based on continuous time:

$$s_{0}=f_{\theta }\left(\{(x_{t_{i}},t_{i}){\}}_{i=1}^{T_{\mathrm{obs}}}\right).$$ (3)

For the purpose of temporal aggregation, the Encoder processes three different categories of features through type-specific embedding layers. Specifically, laboratory values (e.g., hemoglobin, creatinine, albumin) are passed through a fully connected layer along with a batch normalization function. Also, the encoder processes vital sign values separately to maintain their own unique temporal dynamics (e.g., heart rate, blood pressure, oxygen saturation). The encoder creates an embedding for Static Features (e.g., age, sex, body mass index, ASA physical status, surgical procedure category and, comorbidity indices) and concatenates them to the temporal representation of the data from each step in time (e.g., a patient is seen with certain laboratory values at time $t_{i}$, where they have a positional encoding that is based on continuous time $\mathrm{PE}(t_{i})=[\sin\;(\omega_{k}t_{i}),\cos\;(\omega_{k}t_{i}){]}_{k=1}^{d_{\mathrm{pe}}/2}$ and a learned representation of frequencies $\omega_{k}$). Specifically, the encoder allows for a representation of temporal data based on continuous time by learning a unique frequency representation for each temporal data point, enabling the model to accommodate the various time scales for which clinical data are measured (i.e., vital signs generally measured in minutes; laboratory test results measured in hours; and, clinical milestones measured in days). A multi-head attention mechanism (31) is then used to aggregate temporal data using attention weights that are scaled by the time between observations. The method of learned masking is used to account for the fact that there are often many missing values in clinical data (e.g., not all tests are ordered for all patients). Each feature dimension that experiences missing values will have a learned representation for missingness (e.g., the replacement of missing laboratory value(s) with the learned missingness embedding for that feature dimension) as well as a binary indicator of missingness that is concatenated to the feature vector. This allows the model to learn which instances of missingness convey clinical indication without the potential loss of information that could occur with simple imputation (e.g., lack of troponin testing may suggest that low clinical suspicion for an event may exist).

### 4.4 Intervention-conditioned dynamics model

Standardized time models establish the progress of a patient’s condition; however, they cannot address the question that drives preoperative planning: “What happens if an operation is performed differently?” Using $g_{\phi }$, we established a dynamic model that can predict latent state progression based on clinical treatment options. This model allows us to simulate the trajectory of the patient’s condition, allowing us to differentiate DPWM from other standard time predictors.

The recurrent state-space formulation we have adapted to meet the needs of clinical data is based on RSSM as implemented in DreamerV3 (18). In the original RSSM, data is obtained at a fixed rate (e.g., 60 Hz of game frames) with continuous actions taken in limited control spaces. With clinical data there are few characteristics that lead to structural differences between how the model operates and the physician’s data. Specifically: Observations to be processed by the model are collected at irregular intervals and may have missing values across the available preoperative window. The actions taken on patients are actions that can be both temporal in nature (e.g., start/stop medication), and multi-modal (e.g., record inpatient physiotherapy/nutrition activity or procedure timing). There is also a safety constraint that ensures that all trajectories created during simulation will remain within physiologically plausible ranges. The following describes how our dynamics model meets this particular set of conditions:

Unlike fixed time intervals, the dynamics model will compute the transition from one state to another using variable time intervals (Δt)—these are calculated between two clinical events. The neural ordinary differential equation model is being used to determine the deterministic aspect of the transition (as per (35)).

$$h_{t+\Delta t}=h_{t}+\int_{t}^{t+\Delta t}f_{\mathrm{dyn}}(h_{\tau },a_{t})\;d\tau ,$$ (4)

$h_{t}$ is the deterministic recurrent state and $f_{\mathrm{dyn}}$ is a neural network parameterizing the dynamics. Median observed preoperative windows were 2.39 days in MIMIC-IV, 1.62 days in eICU-CRD, and 0.35 days in VitalDB; Day −14 coverage was 1.6%, 0.5%, and 0%, respectively.

Interventions prior to surgery will be represented using an action representation system (**Intervention action encoding.**) that is structured by category of intervention (e.g., medication changes; inpatient physiotherapy/nutrition activity; short-window procedure timing). Each type of intervention receives a separate embedding, which is then combined as part of a composite action vector ($a_{t}=[e_{\mathrm{type}};e_{\mathrm{detail}};\Delta t_{\mathrm{effect}}]$) that includes the type of action embedding, the detailing of the action (e.g., identification of drug, direction of dosage change), and expected time to effect. Each type of intervention is represented in this structured way to allow the dynamics model to learn transition patterns for intervention types; i.e., they will not be treated as alternative input controls.

**Stochastic state component.** The full latent state $s_{t}=(h_{t},z_{t})$ combines the deterministic recurrent state $h_{t}$ with a stochastic component $z_{t}$ sampled from a learned prior:

$$\begin{array}{rl} & z_{t}∼q_{\phi }(z_{t}|h_{t},x_{t})\;\text{( posterior) },\\ & z_{t}∼p_{\phi }(z_{t}|h_{t})\;\text{( prior) }. \end{array}$$ (5)

The posterior probability incorporates data that have been found; however, in counterfactual rollouts where no data exist, the model will sample from the prior distribution. The stochastic component of the model captures the variability for a specific patient as well as epistemic uncertainty; therefore, uncertainty estimates for predicted trajectories that were made are calibrated rather than providing point predictions.

### 4.5 Counterfactual outcome evaluator

The evaluator compares Monte Carlo rollouts of counterfactual outcome estimates, which can then be compared with various treatment strategies based on the variability in patient outcomes associated with the treatment interventions. A Monte Carlo roll-out of a counterfactual outcome estimate will begin with the starting state for a patient at $s_{0}$ and use the clinical intervention sequence of interest ($a_{1\;:\;T}^{\mathrm{cf}}$) to generate K stochastic samples of the outcome trajectory through rolling out the dynamics model.

$${\hat{y}}^{\left(k\right)}=h_{\psi }\left(s_{0\;:\;T}^{\left(k\right)}\right),\;s_{t+1}^{\left(k\right)}=g_{\phi }\left(s_{t}^{\left(k\right)},a_{t}^{\mathrm{cf}}\right),\;k=1,\ldots ,K.$$ (6)

The predicted outcome distribution under the candidate intervention is $P(y|s_{0},a_{1\;:\;T}^{\mathrm{cf}})\approx \frac{1}{K}\sum_{k=1}^{K}{\hat{y}}^{\left(k\right)}$, providing both a point estimate and uncertainty quantification through the empirical variance across rollouts.

$h_{\psi }$ contains two main components: first, a temporal aggregation layer condenses the data into a fixed-length representation (called the trajectory); second, each prediction head creates an individual prediction based on that condensed data (calibrated to match the intended outcome; i.e., all binary classification heads will predict whether or not the patient experienced mortality; individual heads will predict either one of the following: acute kidney injury; sepsis; cardiac event; respiratory failure; or venous thromboembolism; and finally, there is one regression head that predicts how long the patient will stay in the hospital). All classification heads produce probabilities that are calibrated using temperature scaling after being trained (36).

To assess the counterfactual outcome of different interventions, the evaluator calculates the anticipated outcome for each candidate intervention series and ranks them by a composite utility score that balances the benefits of decreased complication vs. the burden of the intervention. For each intervention scenario, we use K=50 rollout samples. These provide stable central tendency estimates (i.e., coefficient of variation <5%) that can also be feasibly calculated in real time.

In order for an intervention sequence to be considered plausible, it cannot produce a physiologically implausible latent state, which would result in out-of-distribution data. That is, if the latent state produced by the counterfactual intervention is outside the convex hull of observed data points, as determined by Mahalanobis distance from the distribution of training samples, that counterfactual will not be included as a recommendation. This conservative approach reflects the principle that counterfactual predictions are valid only “within” (the) support of (the) observed data (28).

### 4.6 Training procedure

DPWM is trained end-to-end on retrospective patient trajectories with documented interventions and known outcomes. The composite loss function comprises four terms:

$$\begin{array}{cc} L & =L_{\mathrm{pred}}+\lambda_{1}L_{\mathrm{dyn}}+\lambda_{2}L_{\mathrm{KL}}+\lambda_{3}L_{\mathrm{recon}},\\ L_{\mathrm{pred}} & =-\sum_{c=1}^{C}\left[y_{c}\log\;{\hat{y}}_{c}+(1-y_{c})\log\;(1-{\hat{y}}_{c})\right],\\ L_{\mathrm{dyn}} & =\sum_{t}\|{\hat{x}}_{t+1}-x_{t+1}{\|}^{2},\\ L_{\mathrm{KL}} & =\sum_{t}D_{\mathrm{KL}}\left(q_{\phi }(z_{t}|h_{t},x_{t})\;\|\;p_{\phi }(z_{t}|h_{t})\right),\\ L_{\mathrm{recon}} & =\sum_{t}\|{\hat{x}}_{t}-x_{t}{\|}^{2}. \end{array}$$ (7)

$L_{\mathrm{pred}}$ is a binary cross-entropy loss between predicted clinical outcomes of C. The dynamics loss $L_{\mathrm{dyn}}$ trains the state transition function (T) so it can predict the next observation, thereby anchoring the latent dynamics in the observable clinical dimensions. The KL divergence term $L_{\mathrm{KL}}$ regularizes the stochastic state component, and therefore prevents posterior collapse whilst maintaining meaningful uncertainty estimates. The reconstruction loss $L_{\mathrm{recon}}$ ensures that the latents retain enough information about the original observations to be able to recover them. Loss weights for each of the losses ($\lambda_{1}=0.5$, $\lambda_{2}=0.1$, $\lambda_{3}=0.3$) are determined by validating the performance of the models on an unseen dataset.

Using the Adam optimizer with hyperparams of $3\times 10^{-4}$ and cosine annealing over 200 epochs at batch size of 64, combined with gradient clipping (norm=1.0) provided stable training of our model(s). Focal loss weighting (γ=2.0) was applied to model prediction loss to account for class imbalance in class predicting, specifically an example using mortality rate <2% in the VitalDB outcome dataset. Each dataset was training complete using an NVIDIA A100 GPU in roughly 8 h of training. We employed early stopping with a patience of 20 epochs based on validation AUROC as a method of preventing overfitting. All hyperparameters were optimized using Bayesian optimization on the validation set; the test dataset was never used during model development.

### 4.7 Datasets and preprocessing

MIMIC-IV was used for model development and temporal internal testing, VitalDB for a separate within-dataset evaluation, and eICU-CRD exclusively for external transfer testing of MIMIC-IV-trained models. All datasets are publicly available through their respective official repositories (VitalDB at vitaldb.net and MIMIC-IV/eICU-CRD on PhysioNet) (24–26), and were downloaded under credentialed access following the standard PhysioNet Data Use Agreement and CITI training requirements (see Data Availability statement for full access procedures).

Among all publicly-available surgical databases, **VitalDB** is the only one that has the ability to record intraoperative data at very high rates with approximately 6,388 surgical cases, it allows for up to 196 different intraoperative monitoring parameters to be stored at both 500 Hz (waveform) and numeric formats every 1 to 7 s, and 73 other perioperative clinical parameters and 34 time-series laboratory results during the 3 months before and after surgery (24). All cases included in this cohort include patients who had undergone non-cardiac surgical procedures at the Seoul National University Hospital (August 2016 to June 2017) and had an in-hospital mortality rate of 0.9% (57 cases). The numeric data is retained as recorded while the waveform data are reduced to minute-based summary statistics (mean/standard deviation/ minimum and maximum per minute). Preoperative features include demographic and comorbidity information and preoperative laboratory results; perioperative trajectory data include a patient’s intraoperative vital signs, medication administration, and fluid management. We utilized an independent 70% of the records to create our training dataset, a separate 15% for validating the model, and an additional 15% for testing using a stratified random method based on the patient’s mortality status and the specialty of the surgeon performing the procedure.

**MIMIC-IV** (VERSION 3.1) contains all surgical procedures and provides data about different types of surgical outcomes. The original **MIMIC-IV** contains 364,627 unique patients with 546,028 hospitalizations (25), who can be categorized as having undergone surgery based on specific ICD procedure codes and admissions types. Approximately 40,000 surgical patients, with 6,000 of these serving as test cases, make up the surgical data in the **MIMIC-IV** database. Included in this data is a variety of preoperative laboratory results (∼160 test types), vital signs, medication administration records, procedure codes, and demographic data. The types of surgical outcomes included in this data are defined as 30-day mortality and 30-day postoperative complications from “acute kidney injury” (AKI), “sepsis”, “cardiac event”, “respiratory failure”, and/or “venous thromboembolism”, through the use of the ICD diagnosis codes for each complication and associated clinical event timestamps. Data was separated into three groups based on when each patient was admitted: patients who were admitted before 2019 were used for training, patients who were admitted during 2019–2020 were used for validation, and patients who were admitted from 2021–2022 were used for testing to avoid temporal leakage of patient information. Additionally, **MIMIC-IV** prohibits the re-use of patients from any one group into another; thus, no patients from the training group were used in the validation or testing groups, respectively.

Across MIMIC-IV and eICU-CRD, postoperative AKI required onset after the indexed procedure or surgical admission. These phenotypes were not independently adjudicated, so cross-database equivalence should not be assumed.

Using **eICU-CRD** (200,859 encounters from 139,367 unique patients across 208 hospitals) as evidence of multi-center generalizability (26), we are able to identify surgical patients through means of APACHE diagnosis codes classified as surgical (i.e., elective or emergent). The database covers 2014–2015, and the full surgical subset (N=18,500) was used only as an external test set for MIMIC-IV-trained models, with follow-up limited to outcomes documented during the indexed encounter.

MIMIC-IV and VitalDB were standardized using their respective training statistics; the external eICU-CRD test set used MIMIC-IV training statistics. Cumulative observations of interventions (i.e., medication given or not given) are taken from medication administration records; physiotherapy/nutrition activity labels were proxies inferred from physiotherapy orders and nutrition-consult timestamps rather than structured multiweek prehabilitation programs.

### 4.8 Baselines and evaluation protocol

We compare DPWM against 14 baselines spanning four architecture families to provide comprehensive evaluation context:

*Traditional clinical scores* may use four different methods: ASA Physical Status classification (3), Portsmouth POSSUM (P-POSSUM), Charlson Comorbidity Index (CCI) (37), and Surgical Outcome Risk Tool (SORT) (4). They represent a wide range of methods for scoring a patient’s risk, with ASA Physical Status classification being completely based on the physician’s opinion and both SORT and CCI being validated multivariate methods (SORT has a c-statistic of 0.91 from its original article).

Three approaches were used by *Static machine learning*: (1) logistic regression with ACS-NSQIP variables (5, 6); (2) Pre-operative EHR features (38) to build an XGBoost model; (3) A deep learning surgical risk prediction model based on DNN architecture (39). These models demonstrated good discrimination (0.83 to 0.946) when applying AUROC metrics but did not incorporate temporal models since all of the features were measured at one point in time.

There are four types of *Temporal deep learning* methods: LSTM (30), temporal convolutional network (TCN) (40), Transformer (31), and Temporal Fusion Transformer (TFT) (11, 32). These models are able to capture sequential dependencies from a medical time series but do not have the ability to condition on interventions or perform counterfactual simulations of the time series data.

*World model and counterfactual methods* utilizes 3 techniques: Foresight (19), G-Transformer (20), and TE-CDE (21). Foresight provides the ability to model EHR timelines without conditioning action (I.e., L1-L2 capabilities). G-Transformer allows for g-computation to support dynamic treatment regime modelling. TE-CDE allows for estimates of counterfactual (from a single intervention) continuous-time outcomes.

Each of the methods utilized receives input features that have been preprocessed in the same manner, and each method is evaluated on the same test set. We use the original code for any published methods where the implementation is officially available and we apply our preprocessing pipeline to that code. Similarly, we utilize the published scoring algorithms for each clinical score, applying them against the extracted feature sets. Published values from different cohorts are presented only as context and are not interpreted as reproduction deviations (Supplementary Table S9). G-Transformer was adapted from a sepsis task and has no external surgical-task anchor; the central comparison therefore depends on our re-implementation.

For the *Evaluation metrics.* Discrimination analysis both AUROC and AUPRC metrics would be calculated, of which AUPRC is more applicable for evaluating models performing poorly with respect to imbalanced class outcomes. Expected calibration error (ECE) (36) is used to assess calibration, with equal bin widths of 10. Clinical applicability will be performed via decision curve analysis (41), which will allow us to compute the net benefit across the different threshold percentage probabilities (1%–50%). Net reclassification improvement (NRI) will represent how well the DPWM model was able to reclassify patients, as compared to the best static baseline model, and componentially (event and non-event) decomposed. For the risk-reclassification analysis, DPWM-predicted 30-day complication probabilities were dichotomized at τ=0.20 (predicted 30-day complication risk ≥20%: high risk; <20%: low risk). The threshold was determined on the validation set, balancing sensitivity, specificity, and the clinically relevant range of the decision curve, and was fixed before the test set and its outcome labels were accessed; it was applied unchanged to all reclassification results (Table 5). Counterfactual concordance was defined as agreement between the model-suggested intervention and the observed decision among patients with favorable outcomes and was therefore descriptive rather than causal. For multiplicity control, one family comprised six MIMIC-IV complication-AUROC comparisons (DPWM vs. XGBoost, DNN, TFT, G-Transformer, TE-CDE, and Foresight); DeLong p-values were adjusted together by the step-down Holm procedure, whereas mortality and external-dataset comparisons were exploratory and unadjusted. All descriptive statistics will be reported with 95% confidence intervals for the computed statistics and all metrics will have bootstrap generated 95% confidence intervals from 1,000 replications using the (10) random seed variance values (42, 123, 256, 389, 512, 678, 741, 853, 927, 1024) derived from the stochastic processes.

Reporting follows TRIPOD+AI, with DECIDE-AI used to frame subsequent live clinical evaluation (42, 43).

### 4.9 Data integrity and leakage prevention

There are rules in place to stop the sharing of information between time, person, and database to keep information secure from leakage. The training, validating, and testing datasets are separated by patient so that no one patient is seen in more than one separate dataset. For the MIMIC-IV database, the testing only has records from patients that are seen after the patients used to create the training set. The standardization for features and statistics of missing values for the features and all preprocessing was determined using only the records from patients used for the training data. eICU-CRD contributed no training or hyperparameter-selection data, whereas VitalDB used its own within-dataset split. Once the preprocessing code has been finalized and cannot be changed before accessing the test data, there will be no further selection of hyperparameters using only the training and validating data. The evaluation of the test data using final model configuration will only occur one time.

## 5 Conclusion

The current study uses data from 3 separate surgical databases to demonstrate how a prior method for estimating a patient’s preoperative risk (static prediction) may be improved by creating a dynamically simulated world model of the patient’s results with clinical treatments as inputs (dynamic trajectory simulation). DPWM provides complication AUROC gains of 1.4–2.2 percentage points over G-Transformer, with the VitalDB comparison not statistically significant. The 76.2% retrospective concordance and risk reclassification results suggest decision-support potential but do not demonstrate causal benefit or clinical optimization.

There are three important points related to perioperative care. First, improvements were larger for complications than for mortality. Second, external calibration deteriorated on eICU-CRD, requiring recalibration before clinical use. Finally, ASA-PS reclassification identifies a subgroup for further evaluation but does not establish benefit from enhanced assessment or short-window inpatient physiotherapy/nutrition activity.

This study indicates that short-term preoperative physiology and recorded care activity can be represented dynamically, but it does not estimate intervention effects. Prospective validation with structured intervention timestamps and established prehabilitation registries is required before clinical use.

## Code availability

Source code, configuration files, and reproducibility scripts are publicly available under the MIT License at https://github.com/ustive/Beyond-Static-Risk-Scores-Dynamic-World-Models. The repository includes environment specifications, tests, verification artifacts, and per-patient test-set prediction outputs for all evaluated models (including the decile-level calibration CSV files) to allow independent verification of the reported metrics; access to the source clinical data remains governed by the original data repositories.

## Data Availability

Publicly available datasets were analyzed in this study. This data can be found here: All datasets analyzed in this study are publicly available via the PhysioNet repository: 1. VitalDB Access link: https://physionet.org/content/vitaldb/1.0.0/ DOI: 10.13026/czw8-9p62 2. MIMIC-IV (version 3.1) Access link: https://physionet.org/content/mimiciv/3.1/ DOI: 10.13026/kpb9-mt58 3. eICU Collaborative Research Database (version 2.0) Access link: https://physionet.org/content/eicu-crd/2.0/ DOI: 10.13026/C2WM1R.
