# Supplementary material for: Beyond static risk scores: dynamic world models simulating patient-specific trajectories to inform preoperative risk mitigation strategies
Source: Front Med (Lausanne). 2026 Sep 17;13:1913206. doi: 10.3389/fmed.2026.1913206 (PMC13627387; doi:10.3389/fmed.2026.1913206)
Supplement: Supplementary file 1 [file Datasheet1.pdf]

1 ADDITIONAL ANALYSES

Table S1: Extended results including AUPRC and ECE for all 15 methods across three databases.

	MIMIC-IV		VitalDB		eICU-CRD	
**Method**	AUPRC	ECE	AUPRC	ECE	AUPRC	ECE
ASA-PS	0.185	0.142	0.048	0.158	0.192	0.148
P-POSSUM	0.225	0.098	0.065	0.112	0.231	0.105
CCI	0.168	0.155	0.042	0.168	0.175	0.160
SORT	0.298	0.075	0.095	0.085	0.305	0.082
ACS-NSQIP (LR)	0.275	0.082	0.082	0.095	0.282	0.088
XGBoost	0.345	0.058	0.118	0.068	0.328	0.055
DNN	0.352	0.062	0.125	0.071	0.335	0.061
LSTM	0.355	0.060	0.128	0.069	0.332	0.063
TCN	0.348	0.063	0.122	0.072	0.325	0.066
Transformer	0.362	0.057	0.130	0.066	0.340	0.060
TFT	0.368	0.055	0.131	0.064	0.342	0.059
Foresight	0.365	0.056	0.129	0.065	0.338	0.060
G-Transformer	0.378	0.052	0.136	0.061	0.349	0.058
TE-CDE	0.372	0.054	0.133	0.063	0.345	0.059
**DPWM**	**0.398**	**0.048**	**0.142**	**0.059**	**0.358**	0.072

Table S2: Pairwise ablation interactions on MIMIC-IV complication AUROC. Diagonal: single-component removal. Off-diagonal: simultaneous removal of two components. IR (interaction ratio) > 1.0 indicates synergy.

	Dynamics	Interv. Cond.	Counterfact.	Temp. Attn.
Dynamics	0.843	0.835 (IR=1.15)	0.838	0.830
Interv. Cond.	—	0.868	0.870	0.856
Counterfact.	—	—	0.876	0.862
Temp. Attn.	—	—	—	0.865

1.1 Patient-level and candidate-rollout diagnostics

The following diagnostics were computed from the patient-level and candidate-rollout outputs of the study experiments.

In the eICU-CRD sample ($N = 18{,}500$), 10-bin ECE was 0.072 for DPWM, 0.058 for G-Transformer, and 0.055 for XGBoost, consistent with the observed direction of external calibration differences in the accompanying decile-level CSV. Within the ASA I–II subgroup, the reclassification ORs were 2.75 (95% CI 2.06–3.68) for DPWM, 3.69 (2.80–4.86) for DNN, and 3.07 (2.31–4.08) for XGBoost; this analysis cannot establish comparative subgroup performance. Mahalanobis support retained 95.0% of rollouts overall and 96.9%, 95.2%, and 91.5% for medication, PT/nutrition, and procedure-timing candidates, respectively. Median preoperative windows were 2.39 days (IQR 1.36–4.15) for MIMIC-IV, 1.62 (0.92–2.85) for eICU-CRD, and 0.35 (0.19–0.62) for VitalDB; corresponding Day $-7/-14$ coverage was 9.8%/1.6%, 4.2%/0.5%, and 0%/0%, respectively.

Table S3: Hyperparameter sensitivity analysis on MIMIC-IV and VitalDB (complication AUROC). Default configuration in **bold**. Best per-dataset configuration marked with $\star$.

Hyperparameter	Value	MIMIC-IV	VitalDB	eICU-CRD	Δ MIMIC	Δ VitalDB
Latent dim (d_s)	64	0.871	0.828	0.848	−1.2	−1.0
	**128**	**0.883**	**0.838**	**0.859**	—	—
	256	0.885$\star$	0.835	0.857	+0.2	–0.3
	512	0.881	0.840$\star$	0.854	–0.2	+0.2
Rollout samples (K)	10	0.878	0.833	0.855	–0.5	–0.5
	25	0.881	0.836	0.857	–0.2	–0.2
	**50**	**0.883**	**0.838**	**0.859**	—	—
	100	0.884	0.839	0.859	+0.1	+0.1
Learning rate	1×10^{-4}	0.877	0.834	0.854	–0.6	–0.4
	3×10^{-4}	**0.883**	**0.838**	**0.859**	—	—
	1×10^{-3}	0.879	0.832	0.852	–0.4	–0.6
	3×10^{-3}	0.862	0.815	0.838	–2.1	–2.3
λ_{dyn}	0.1	0.876	0.831	0.852	–0.7	–0.7
	**0.5**	**0.883**	**0.838**	**0.859**	—	—
	1.0	0.880	0.836	0.856	–0.3	–0.2

Table S4: Representative failure cases from retrospective validation ($N = 53$ discordant cases). Each row is a representative example from the corresponding failure mechanism category.

Category	Case Example	Predicted	Actual	Root Cause
Data quality	MIMIC-IV, 68M, hip replacement	Low risk	AKI Stage 2	Missing preop creatinine, albumin
Rare complication	VitalDB, 45F, appendectomy	Low risk	Postop DVT	VTE base rate 1.2%; insufficient training signal
Distribution shift	eICU-CRD, 72M, emergency laparotomy	Moderate risk	Died day 3	Rural facility; different monitoring protocols
Atypical presentation	MIMIC-IV, 32F, cesarean section	Low risk	Severe sepsis	Atypical postpartum sepsis, no preop indicators
Overconfidence	MIMIC-IV, 58M, cardiac surgery	High confidence low risk	Postop AF	Model predicted 8% AF probability; actual rate 24%

Table S5: Dataset statistics and outcome distributions. VitalDB's 0.9% test mortality corresponds to nine deaths; mortality discrimination is not reported.

			Complication Rate (%)						
**Dataset**	N_{test}	**Mortality (%)**	AKI	Sepsis	Cardiac	Resp.	VTE	**Features**	**Median Obs/Patient**
VitalDB	958	0.9	3.8	1.5	2.1	2.8	1.2	303	2,800,000
MIMIC-IV	6,000	3.2	8.5	5.2	4.8	6.1	2.3	∼160	1,250
eICU-CRD	18,500	3.8	7.2	4.8	3.9	5.5	2.0	∼120	580

Test-split event counts (n): VitalDB — mortality 9, AKI 36, sepsis 14, cardiac 20, respiratory 27, VTE 11; MIMIC-IV — mortality 192, AKI 510, sepsis 312, cardiac 288, respiratory 366, VTE 138; eICU-CRD — mortality 703, AKI 1,332, sepsis 888, cardiac 722, respiratory 1,018, VTE 370.

Table S6: Per-complication AUROC breakdown on MIMIC-IV for DPWM and top baselines.

Method	AKI	Sepsis	Cardiac	Respiratory	VTE
XGBoost	0.852	0.821	0.835	0.828	0.778
DNN	0.858	0.832	0.842	0.835	0.785
TFT	0.872	0.848	0.856	0.852	0.802
G-Transformer	0.878	0.852	0.861	0.858	0.808
**DPWM**	**0.905**	**0.878**	**0.882**	**0.875**	0.815
Δ (DPWM − G-Transf.)	+2.7	+2.6	+2.1	+1.7	+0.7

Table S7: Seed variance analysis for DPWM across 10 random seeds on MIMIC-IV. CV% = coefficient of variation. Worst seed must exceed best baseline mean (G-Transformer).

Metric	Mean	Std	CV%	Best Seed	Worst Seed	Best Baseline Mean
Mortality AUROC	0.918	0.008	0.87	0.931 (seed 389)	0.904 (seed 853)	0.895 (G-Transf.)
Complication AUROC	0.883	0.010	1.13	0.899 (seed 741)	0.868 (seed 256)	0.861 (G-Transf.)
ECE	0.048	0.006	12.5	0.039 (seed 123)	0.058 (seed 512)	0.052 (G-Transf.)
AUPRC	0.398	0.015	3.77	0.422 (seed 927)	0.375 (seed 42)	0.378 (G-Transf.)

Table S7b: Statistical significance of DPWM vs. baselines (DeLong test, MIMIC-IV complication AUROC). Holm-Bonferroni-corrected p-values were computed within one family of the six primary MIMIC-IV complication-AUROC comparisons using the step-down procedure (multipliers $6, 5, 4, 3, 2, 1$ with cumulative maximum). Conservative bounds ($p < 0.006$) are reported where raw values were available only as $p < 0.001$; exploratory comparisons are unadjusted.

Comparison	Δ AUROC	p (uncorr.)	p (H-B corr.)	Type
DPWM vs. XGBoost	+0.051	<0.001	<0.006	Primary
DPWM vs. DNN	+0.042	<0.001	<0.006	Primary
DPWM vs. TFT	+0.025	0.003	0.006	Primary
DPWM vs. G-Transformer	+0.022	0.005	0.006	Primary
DPWM vs. TE-CDE	+0.028	0.002	0.006	Primary
DPWM vs. Foresight	+0.031	0.001	<0.006	Primary
DPWM vs. Transformer	+0.032	0.001	—	Exploratory
DPWM vs. LSTM	+0.038	<0.001	—	Exploratory
DPWM vs. TCN	+0.045	<0.001	—	Exploratory

Table S8: Full statistical details for primary comparisons. Effect size (Cohen's d), 95% bootstrap CI, and power analysis. MIMIC-IV rows report Holm-adjusted p-values within the six-comparison family (Table S7b); VitalDB and eICU-CRD rows are exploratory and report unadjusted p-values.

Dataset	Comparison	Δ AUROC	Cohen's d	95% CI	p (H-B)	Power
MIMIC-IV	DPWM vs. XGBoost (comp.)	+0.051	0.72	[0.032, 0.070]	<0.006	0.94
MIMIC-IV	DPWM vs. G-Transformer (comp.)	+0.022	0.38	[0.006, 0.038]	0.006	0.78
MIMIC-IV	DPWM vs. TFT (comp.)	+0.025	0.42	[0.009, 0.041]	0.006	0.81
VitalDB	DPWM vs. XGBoost (comp.)	+0.037	0.45	[0.008, 0.066]	0.018	0.62
VitalDB	DPWM vs. G-Transformer (comp.)	+0.014	0.18	[−0.012, 0.040]	0.298	0.28
eICU-CRD	DPWM vs. XGBoost (comp.)	+0.044	0.58	[0.025, 0.063]	<0.001	0.91
eICU-CRD	DPWM vs. G-Transformer (comp.)	+0.018	0.26	[0.001, 0.035]	0.042	0.54

Table S9: Baseline implementation provenance. Published values from different cohorts are contextual and are not interpreted as reproduction error; G-Transformer has no published surgical-task anchor.

Baseline	Dataset	Published	Our Reprod.	Code Source	
XGBoost	MIMIC-IV (mort.)	0.9376	0.901	Lee GitHub	2022
TFT	MIMIC-IV (mort.)	0.91 (int.)	0.892	Kapral GitHub	2024
G-Transformer	MIMIC-IV (comp.)	N/A (new task)	0.861	Xiong GitHub	2024

Note: Published and study values use different cohorts, endpoints, preprocessing, or splits and are not reproduction deviations; no cross-cohort delta is computed.
G-Transformer: Official code was adapted from a sepsis cohort; no external surgical benchmark is available.

Table S10: Expanded retrospective validation by outcome type and patient subgroup ($N = 250$).

Subgroup	N	Concordance (%)	κ	Sensitivity	Specificity
By outcome type:					
Mortality	45	84.4	0.68	0.81	0.86
AKI	52	80.8	0.64	0.78	0.83
Sepsis	38	76.3	0.58	0.74	0.78
Cardiac events	35	77.1	0.59	0.75	0.79
Respiratory	42	78.6	0.61	0.76	0.81
No complication	38	81.6	0.65	0.83	0.80
By ASA class:					
ASA I–II	85	82.4	0.66	0.80	0.84
ASA III	98	78.6	0.62	0.76	0.81
ASA IV+	67	73.1	0.54	0.71	0.75
By database:					
VitalDB	60	78.3	0.58	0.76	0.80
MIMIC-IV	130	82.3	0.67	0.80	0.84
eICU-CRD	60	72.1	0.52	0.70	0.74
